# Correction: Obesity-related indicators and tuberculosis: A Mendelian randomization study

**DOI:** 10.1371/journal.pone.0316856

**Published:** 2024-12-30

**Authors:** Nuannuan Cai, Weiyan Luo, Lili Ding, Lijin Chen, Yuanjiang Huang

There is an error in affiliation 1 for authors Nuannuan Cai, Weiyan Luo, Lili Ding and Lijin Chen. The correct affiliation 1 is: Pulmonary and Critical Care Medicine, Hainan General Hospital, Hainan Affiliate Hospital Of Hainan Medical University, Haikou, Hainan, China.
