# Supplementary figures and images for: Obesity-related indicators and tuberculosis: A Mendelian randomization study
Source: PLoS One. 2024 Apr 1;19(4):e0297905. doi: 10.1371/journal.pone.0297905 (PMC10984409; doi:10.1371/journal.pone.0297905)

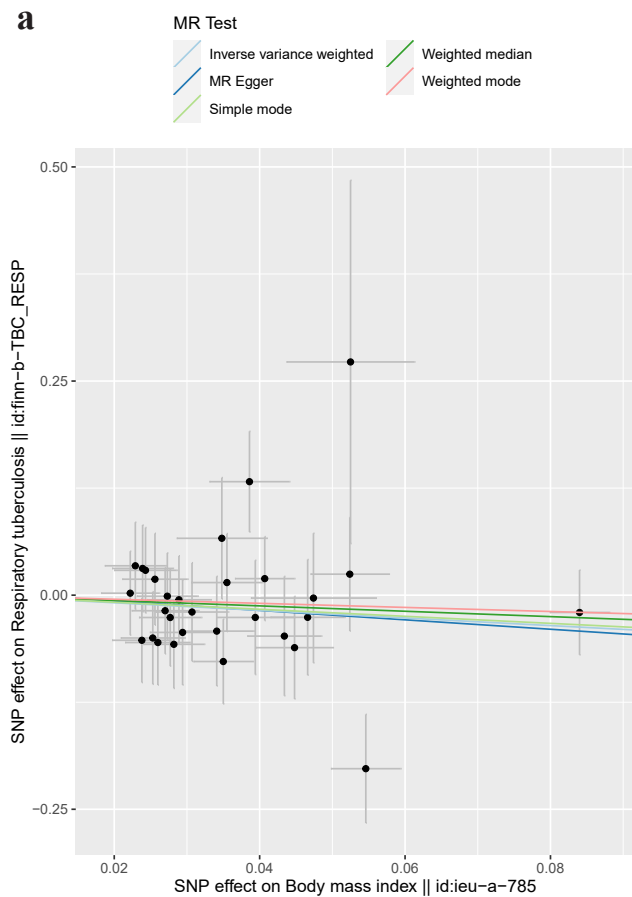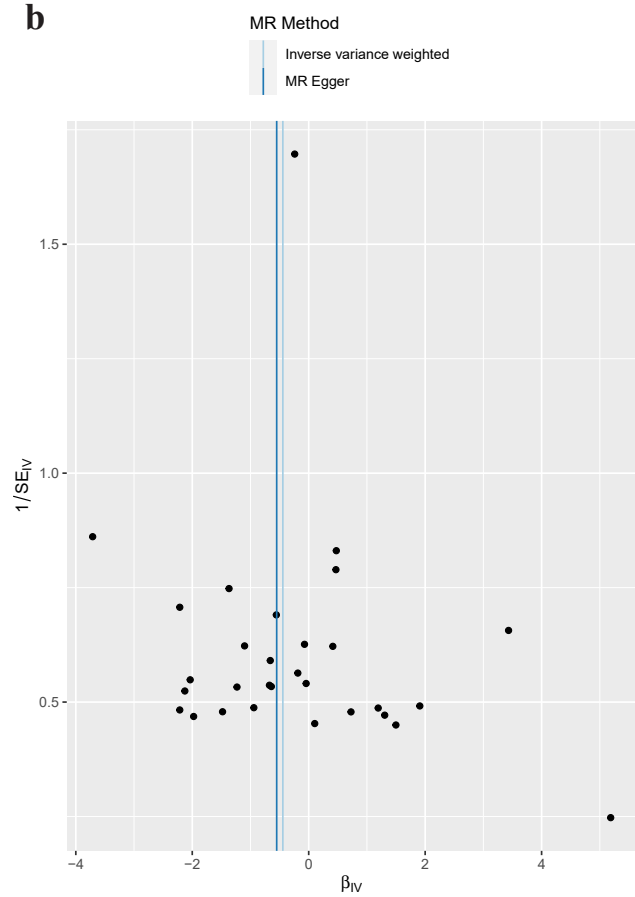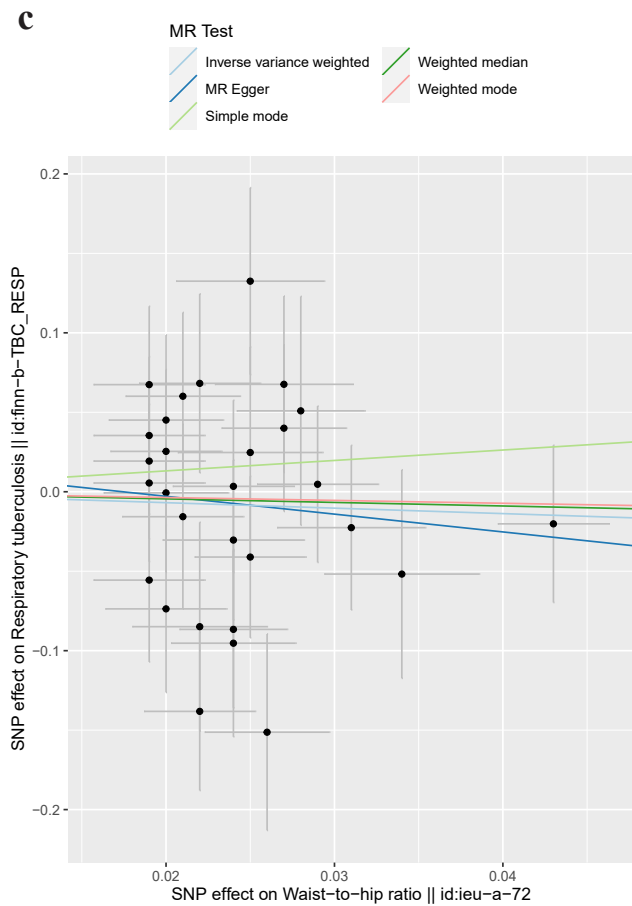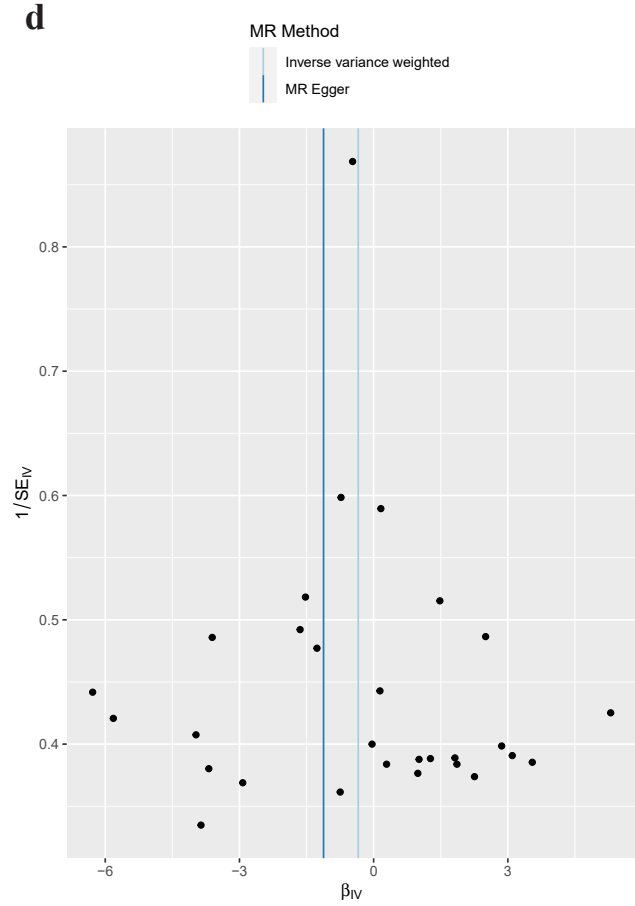

Supplement: S1 Fig — (a, c): Scatter and funnel plots of body mass index. (c, d): Scatter and funnel plot of waist-hip ratio. (PDF) [file pone.0297905.s001.pdf]
