# Supplementary material for: Obesity-related indicators and tuberculosis: A Mendelian randomization study
Source: PLoS One. 2024 Apr 1;19(4):e0297905. doi: 10.1371/journal.pone.0297905 (PMC10984409; doi:10.1371/journal.pone.0297905)
Supplement: S1 Table — (DOCX) [file pone.0297905.s002.docx]

**S1 Table: Harmonized dataset of Mendelian randomization for the effect of body mass index on respiratory tuberculosis.**

| **SNP** | **Effect allele** | **Other allele** | **Chr** | **Exposure** | | | **Outcome** | | |
| --- | --- | --- | --- | --- | --- | --- | --- | --- | --- |
|  |  |  |  | ***β*** | **SE** | ***p*** | ***β*** | **SE** | ***p*** |
| **rs10132280** | A | C | 14 | -0.0289 | 0.0044 | 4.96E-11 | 0.000221963 | 0.000159526 | 0.16 |
| **rs11209963** | G | A | 1 | 0.0434 | 0.0051 | 3.37E-17 | 0.000121404 | 0.000183714 | 0.51 |
| **rs11604680** | G | A | 11 | 0.0277 | 0.0043 | 1E-10 | -7.94E-05 | 1.56E-04 | 6.10E-01 |
| **rs11676272** | G | A | 2 | 0.026 | 0.0045 | 5.23E-09 | 1.53E-05 | 1.46E-04 | 9.20E-01 |
| **rs1222069** | C | A | 1 | -0.0243 | 0.0043 | 1.05E-08 | 0.000115768 | 0.000151044 | 0.44 |
| **rs12286929** | G | A | 11 | 0.0222 | 0.004 | 3.55E-08 | 0.000197287 | 0.000145738 | 0.18 |
| **rs12429545** | A | G | 13 | 0.0348 | 0.0062 | 1.51E-08 | 0.000106663 | 0.000218358 | 0.630001 |
| **rs13107325** | T | C | 4 | 0.0525 | 0.0088 | 2.72E-09 | -1.31E-05 | 2.82E-04 | 9.60E-01 |
| **rs13130484** | T | C | 4 | 0.0407 | 0.0041 | 6.05E-23 | 1.15E-05 | 1.47E-04 | 9.40E-01 |
| **rs1421085** | C | T | 16 | 0.084 | 0.0041 | 4.11E-94 | 0.000120538 | 0.00014939 | 0.42 |
| **rs1528435** | T | C | 2 | 0.0229 | 0.0042 | 4.32E-08 | -0.000189249 | 0.000149798 | 0.21 |
| **rs16907751** | T | C | 8 | -0.0474 | 0.0086 | 3.89E-08 | 0.000392653 | 0.00024471 | 0.11 |
| **rs17094222** | C | T | 10 | 0.0307 | 0.005 | 8.02E-10 | -0.000285098 | 0.000179567 | 0.11 |
| **rs17109256** | A | G | 14 | 0.0355 | 0.0049 | 7.14E-13 | -0.000250545 | 0.000176347 | 0.16 |
| **rs2176040** | G | A | 2 | -0.0239 | 0.0042 | 9.99E-09 | -6.49E-05 | 1.52E-04 | 6.70E-01 |
| **rs2206277** | T | C | 6 | 0.0448 | 0.0053 | 1.69E-17 | 0.000197467 | 0.00018907 | 0.3 |
| **rs2287019** | T | C | 19 | -0.0386 | 0.0055 | 2.69E-12 | -0.000159023 | 0.000189761 | 0.4 |
| **rs253414** | T | C | 5 | 0.027 | 0.0046 | 3.92E-09 | 0.000126993 | 0.000154223 | 0.41 |
| **rs2820315** | T | C | 1 | 0.0253 | 0.0044 | 6.63E-09 | -1.65E-05 | 1.57E-04 | 9.20E-01 |
| **rs3888190** | A | C | 16 | 0.035 | 0.0041 | 1.32E-17 | -0.000167992 | 0.000149259 | 0.26 |
| **rs4776970** | T | A | 15 | -0.0282 | 0.0042 | 1.89E-11 | 0.00024306 | 0.000151181 | 0.11 |
| **rs492400** | T | C | 2 | -0.0238 | 0.0041 | 6.78E-09 | -0.00027377 | 0.000147 | 0.0629999 |
| **rs543874** | G | A | 1 | 0.0341 | 0.0051 | 2.73E-11 | -0.000124603 | 0.000180016 | 0.49 |
| **rs6265** | T | C | 11 | -0.0466 | 0.0051 | 1.09E-19 | -0.000286794 | 0.000186729 | 0.12 |
| **rs6567160** | C | T | 18 | 0.0546 | 0.0048 | 4.55E-30 | -0.00015498 | 0.000171614 | 0.37 |
| **rs6734363** | A | G | 2 | 0.0524 | 0.0054 | 2.01E-22 | 1.71E-05 | 1.93E-04 | 9.30E-01 |
| **rs7138803** | A | G | 12 | 0.0273 | 0.0042 | 5.9E-11 | -6.43E-05 | 1.51E-04 | 6.70E-01 |
| **rs7599312** | A | G | 2 | -0.0256 | 0.0045 | 8.68E-09 | -0.000100829 | 0.000166089 | 0.54 |
| **rs7903146** | T | C | 10 | -0.0294 | 0.0045 | 3.89E-11 | 4.52E-06 | 1.60E-04 | 9.80E-01 |
| **rs9816226** | T | A | 3 | 0.0394 | 0.0054 | 3.94E-13 | -0.000356293 | 0.000187749 | 0.0580003 |

Chr: Chromosome.
