# Supplementary material for: Obesity-related indicators and tuberculosis: A Mendelian randomization study
Source: PLoS One. 2024 Apr 1;19(4):e0297905. doi: 10.1371/journal.pone.0297905 (PMC10984409; doi:10.1371/journal.pone.0297905)
Supplement: S2 Table — (DOCX) [file pone.0297905.s003.docx]

**S2 Table: Harmonized dataset of Mendelian randomization for the effect of waist-to-hip ratio on respiratory tuberculosis.**

| **SNP** | **Effect allele** | **Other allele** | **Chr** | **Exposure** | | | **Outcome** | | |
| --- | --- | --- | --- | --- | --- | --- | --- | --- | --- |
|  |  |  |  | ***β*** | **SE** | ***p*** | ***β*** | **SE** | ***p*** |
| **rs10245353** | A | C | 7 | 0.027 | 0.0041 | 3.50E-11 | -4.08E-05 | 0.000185522 | 0.83 |
| **rs10783615** | A | G | 12 | -0.034 | 0.0046 | 3.70E-13 | -0.000129951 | 0.000210629 | 0.54 |
| **rs10842708** | A | G | 12 | -0.024 | 0.0036 | 3.10E-11 | -0.00021562 | 0.000168482 | 0.2 |
| **rs1106529** | A | G | 1 | 0.028 | 0.0038 | 2.20E-13 | -3.62E-05 | 0.000165493 | 0.83 |
| **rs1128249** | T | G | 2 | -0.02 | 0.0033 | 7.80E-10 | -0.000107578 | 0.000149528 | 0.47 |
| **rs11663816** | C | T | 18 | 0.026 | 0.0037 | 3.10E-12 | -0.000140418 | 0.000165168 | 0.4 |
| **rs12549058** | G | T | 8 | 0.037 | 0.006 | 8.30E-10 | -0.000314328 | 0.000298722 | 0.29 |
| **rs1294410** | C | T | 6 | 0.025 | 0.0033 | 2.00E-14 | 4.99E-05 | 0.000150585 | 0.74 |
| **rs13130484** | T | C | 4 | 0.019 | 0.0033 | 1.50E-08 | 1.15E-05 | 0.000147253 | 0.94 |
| **rs13424740** | C | T | 2 | -0.024 | 0.0032 | 2.60E-13 | -7.87E-05 | 0.000146499 | 0.59 |
| **rs1421085** | C | T | 16 | 0.043 | 0.0033 | 4.30E-38 | 0.000120538 | 0.00014939 | 0.42 |
| **rs1440372** | C | T | 15 | 0.022 | 0.0036 | 2.70E-09 | 8.21E-05 | 0.00016421 | 0.62 |
| **rs1563355** | C | T | 1 | 0.031 | 0.0044 | 1.70E-12 | 9.69E-05 | 0.000154562 | 0.53 |
| **rs16996700** | C | T | 20 | -0.021 | 0.0036 | 8.80E-09 | -0.000184384 | 0.00016124 | 0.25 |
| **rs17451107** | C | T | 3 | -0.021 | 0.0034 | 1.20E-09 | 1.35E-05 | 0.000149753 | 0.93 |
| **rs2287019** | T | C | 19 | -0.025 | 0.0044 | 1.10E-08 | -0.000159023 | 0.000189761 | 0.4 |
| **rs2371767** | C | G | 3 | -0.024 | 0.0037 | 1.20E-10 | 0.000180632 | 0.000162084 | 0.27 |
| **rs2398893** | G | A | 9 | -0.02 | 0.0036 | 4.00E-08 | 8.34E-05 | 0.000162589 | 0.61 |
| **rs2972164** | C | T | 3 | 0.019 | 0.0033 | 1.00E-08 | -1.28E-05 | 0.000147122 | 0.93 |
| **rs3786897** | G | A | 19 | 0.022 | 0.0033 | 2.50E-11 | -2.44E-05 | 0.000147281 | 0.87 |
| **rs459193** | G | A | 5 | -0.027 | 0.0037 | 3.40E-13 | 2.66E-05 | 0.000165647 | 0.87 |
| **rs4640244** | G | A | 17 | 0.02 | 0.0037 | 3.00E-08 | 8.62E-05 | 0.000149163 | 0.56 |
| **rs4823006** | G | A | 22 | -0.019 | 0.0033 | 3.30E-09 | 1.59E-05 | 0.000146016 | 0.91 |
| **rs4929927** | G | A | 11 | 0.019 | 0.0033 | 1.60E-08 | -0.000228247 | 0.000152225 | 0.13 |
| **rs6736025** | T | G | 2 | -0.024 | 0.0042 | 7.20E-09 | -9.18E-05 | 0.000146441 | 0.53 |
| **rs6743060** | A | C | 2 | 0.025 | 0.0043 | 1.50E-08 | 1.39E-05 | 0.000193632 | 0.94 |
| **rs714515** | A | G | 1 | -0.019 | 0.0033 | 5.90E-09 | 0.000189016 | 0.000147521 | 0.2 |
| **rs7973683** | A | C | 12 | -0.02 | 0.0034 | 3.30E-09 | 7.87E-05 | 0.000153815 | 0.61 |
| **rs879048** | A | C | 11 | 0.022 | 0.004 | 3.60E-08 | 0.000297852 | 0.000179425 | 0.0969996 |
| **rs9491696** | G | C | 6 | 0.037 | 0.0032 | 1.40E-30 | 6.90E-05 | 0.000145327 | 0.64 |
| **rs998584** | A | C | 6 | 0.029 | 0.0036 | 4.80E-15 | -0.000117072 | 0.000146371 | 0.42 |

Chr: Chromosome.
