# Supplementary material for: Obesity-related indicators and tuberculosis: A Mendelian randomization study
Source: PLoS One. 2024 Apr 1;19(4):e0297905. doi: 10.1371/journal.pone.0297905 (PMC10984409; doi:10.1371/journal.pone.0297905)
Supplement: S3 Table — (DOCX) [file pone.0297905.s004.docx]

**S3 Table: Harmonized dataset of Mendelian randomization for the effect of hip circumference on respiratory tuberculosis.**

| **SNP** | **Effect allele** | **Other allele** | **Chr** | **Exposure** | | | **Outcome** | | |
| --- | --- | --- | --- | --- | --- | --- | --- | --- | --- |
|  |  |  |  | ***β*** | **SE** | ***p*** | ***β*** | **SE** | ***p*** |
| **rs10123368** | C | T | 9 | 0.025 | 0.0043 | 3.30E-09 | 0.0001846 | 0.000184 | 0.32 |
| **rs10140922** | T | G | 14 | -0.03 | 0.0046 | 4.60E-11 | -0.000314 | 0.000148 | 0.034 |
| **rs10195252** | C | T | 2 | 0.023 | 0.0035 | 7.80E-11 | -0.00018 | 0.000149 | 0.23 |
| **rs1046934** | C | A | 1 | 0.022 | 0.0037 | 8.30E-10 | -4.99E-05 | 0.000153 | 0.74 |
| **rs1053593** | T | G | 22 | 0.021 | 0.0037 | 2.80E-08 | 0.0002532 | 0.000153 | 0.097 |
| **rs10748128** | T | G | 12 | 0.025 | 0.0038 | 4.90E-11 | 6.83E-05 | 0.000152 | 0.649999 |
| **rs10804591** | A | C | 3 | -0.037 | 0.0042 | 1.10E-18 | -6.46E-05 | 0.000176 | 0.709999 |
| **rs10950949** | C | A | 7 | -0.021 | 0.0036 | 1.50E-08 | 8.05E-05 | 0.000148 | 0.59 |
| **rs10958476** | C | T | 8 | 0.027 | 0.0045 | 1.20E-09 | 0.0001724 | 0.000179 | 0.34 |
| **rs11144688** | A | G | 9 | -0.035 | 0.0063 | 2.70E-08 | 0.0001819 | 0.000226 | 0.42 |
| **rs11205303** | C | T | 1 | 0.042 | 0.004 | 6.40E-26 | 0.0002073 | 0.000149 | 0.16 |
| **rs11242** | C | T | 3 | -0.024 | 0.0035 | 2.60E-12 | 0.0001975 | 0.000147 | 0.18 |
| **rs11612228** | T | C | 12 | 0.023 | 0.004 | 8.60E-09 | 0.0001218 | 0.000156 | 0.43 |
| **rs1173770** | C | T | 5 | -0.026 | 0.0035 | 1.30E-13 | -0.000197 | 0.000149 | 0.19 |
| **rs12048049** | G | C | 1 | 0.023 | 0.0038 | 1.20E-09 | 0.0001578 | 0.00016 | 0.32 |
| **rs12086130** | T | C | 1 | 0.038 | 0.006 | 1.70E-10 | 0.0004105 | 0.000252 | 0.1 |
| **rs12130243** | G | T | 1 | -0.029 | 0.0051 | 1.90E-08 | -0.000268 | 0.000181 | 0.14 |
| **rs12316131** | C | T | 12 | 0.032 | 0.0042 | 3.30E-14 | 9.51E-05 | 0.000179 | 0.6 |
| **rs1254257** | C | T | 14 | 0.029 | 0.0048 | 9.10E-10 | 9.76E-05 | 0.000159 | 0.54 |
| **rs12817549** | C | T | 12 | -0.028 | 0.0035 | 1.10E-15 | 5.89E-05 | 0.000146 | 0.69 |
| **rs12980348** | G | T | 19 | 0.028 | 0.0036 | 3.50E-15 | -0.000104 | 0.000151 | 0.49 |
| **rs1351394** | C | T | 12 | -0.024 | 0.0034 | 9.60E-13 | 0.0002542 | 0.000146 | 0.081999 |
| **rs13695** | T | C | 17 | 0.027 | 0.0044 | 6.00E-10 | -0.000338 | 0.000167 | 0.043 |
| **rs1388251** | G | A | 3 | -0.023 | 0.0039 | 1.80E-09 | -9.72E-05 | 0.000165 | 0.56 |
| **rs143384** | G | A | 20 | 0.044 | 0.0037 | 2.80E-32 | 0.0002128 | 0.000149 | 0.15 |
| **rs1545552** | G | A | 2 | 0.03 | 0.004 | 8.50E-14 | -6.80E-05 | 0.000162 | 0.67 |
| **rs1662837** | C | T | 4 | 0.027 | 0.0037 | 3.60E-13 | 0.0002026 | 0.000159 | 0.2 |
| **rs16894959** | C | T | 6 | 0.035 | 0.0048 | 2.90E-13 | 9.50E-05 | 0.000209 | 0.649999 |
| **rs17194040** | A | G | 16 | 0.024 | 0.004 | 6.20E-10 | -0.000214 | 0.000163 | 0.19 |
| **rs17346473** | G | A | 1 | 0.03 | 0.0039 | 2.60E-14 | 2.76E-05 | 0.000162 | 0.87 |
| **rs17819328** | G | T | 3 | -0.022 | 0.0036 | 5.20E-10 | -0.000333 | 0.000148 | 0.025 |
| **rs1812175** | G | A | 4 | 0.054 | 0.0046 | 1.20E-31 | 0.0003036 | 0.000193 | 0.11 |
| **rs181553** | A | G | 18 | 0.028 | 0.0037 | 2.10E-14 | 0.0001839 | 0.000158 | 0.24 |
| **rs2034088** | C | T | 17 | -0.021 | 0.0035 | 1.30E-09 | -0.00025 | 0.000147 | 0.089 |
| **rs2247341** | A | G | 4 | 0.023 | 0.0036 | 2.70E-10 | 0.0001045 | 0.000154 | 0.5 |
| **rs2326788** | A | G | 20 | -0.02 | 0.0036 | 2.00E-08 | 5.65E-05 | 0.000151 | 0.709999 |
| **rs2377058** | G | A | 16 | 0.023 | 0.0037 | 7.70E-10 | -0.000286 | 0.000151 | 0.058 |
| **rs2597513** | T | C | 3 | -0.033 | 0.0057 | 8.40E-09 | 2.62E-06 | 0.000238 | 0.99 |
| **rs2638953** | C | G | 12 | 0.023 | 0.0037 | 2.90E-10 | -9.25E-05 | 0.000156 | 0.55 |
| **rs2820443** | C | T | 1 | 0.047 | 0.0038 | 2.80E-35 | -8.28E-05 | 0.00016 | 0.61 |
| **rs3118914** | T | G | 13 | -0.032 | 0.0042 | 1.70E-14 | 7.99E-05 | 0.000179 | 0.66 |
| **rs3738814** | G | A | 1 | -0.03 | 0.0035 | 1.60E-17 | -0.000144 | 0.000147 | 0.33 |
| **rs3747579** | T | C | 16 | 0.022 | 0.0039 | 1.30E-08 | 7.92E-05 | 0.000161 | 0.62 |
| **rs3791679** | G | A | 2 | -0.04 | 0.0041 | 2.00E-22 | -0.000359 | 0.000174 | 0.039 |
| **rs4130415** | C | T | 8 | -0.037 | 0.0043 | 5.90E-18 | 0.0001068 | 0.000172 | 0.53 |
| **rs42235** | T | C | 7 | 0.033 | 0.0038 | 1.60E-18 | 5.71E-05 | 0.000159 | 0.719999 |
| **rs4239437** | C | T | 18 | 0.034 | 0.0043 | 3.70E-15 | 0.0002166 | 0.000179 | 0.23 |
| **rs4243400** | G | A | 3 | 0.023 | 0.0035 | 3.30E-11 | -0.000135 | 0.000146 | 0.36 |
| **rs4246307** | A | G | 15 | -0.024 | 0.0043 | 4.20E-08 | 3.82E-05 | 0.000146 | 0.79 |
| **rs4620037** | C | A | 5 | -0.024 | 0.0043 | 2.40E-08 | -0.000278 | 0.000178 | 0.12 |
| **rs473902** | G | T | 9 | -0.057 | 0.0075 | 3.40E-14 | 3.84E-05 | 0.000261 | 0.88 |
| **rs4868126** | G | T | 5 | 0.02 | 0.0036 | 1.50E-08 | -1.51E-05 | 0.000153 | 0.92 |
| **rs4973517** | C | T | 2 | -0.027 | 0.0044 | 9.30E-10 | -0.000225 | 0.000163 | 0.17 |
| **rs5742915** | C | T | 15 | 0.02 | 0.0036 | 1.70E-08 | 4.28E-05 | 0.000147 | 0.77 |
| **rs606452** | C | A | 11 | -0.028 | 0.0049 | 9.30E-09 | 0.0001942 | 0.000206 | 0.35 |
| **rs6501392** | G | A | 17 | 0.021 | 0.0034 | 3.90E-10 | 0.0002266 | 0.000146 | 0.12 |
| **rs6556079** | A | G | 5 | -0.028 | 0.0045 | 4.50E-10 | -3.71E-05 | 0.000149 | 0.8 |
| **rs6570509** | T | G | 6 | -0.043 | 0.0039 | 9.40E-29 | 3.70E-05 | 0.00016 | 0.82 |
| **rs6845078** | T | C | 4 | -0.033 | 0.0048 | 6.40E-12 | -7.79E-06 | 0.000196 | 0.97 |
| **rs686320** | C | G | 11 | -0.031 | 0.0053 | 4.10E-09 | -0.00016 | 0.00021 | 0.450001 |
| **rs6903448** | T | C | 6 | -0.035 | 0.0047 | 1.90E-13 | -0.000263 | 0.000205 | 0.2 |
| **rs7008867** | A | G | 8 | 0.023 | 0.004 | 4.70E-09 | 9.74E-06 | 0.000175 | 0.96 |
| **rs7162542** | G | C | 15 | 0.039 | 0.0035 | 4.30E-29 | -0.000223 | 0.000148 | 0.13 |
| **rs7187776** | G | A | 16 | 0.02 | 0.0035 | 1.50E-08 | -0.000167 | 0.000149 | 0.26 |
| **rs7223966** | A | G | 17 | 0.028 | 0.0038 | 1.60E-13 | -0.000203 | 0.000162 | 0.21 |
| **rs724016** | G | A | 3 | 0.048 | 0.0035 | 8.00E-43 | 0.0002241 | 0.000147 | 0.13 |
| **rs7274811** | T | G | 20 | -0.024 | 0.004 | 1.30E-09 | 4.62E-07 | 0.000166 | 1 |
| **rs7759938** | T | C | 6 | -0.025 | 0.0037 | 5.20E-12 | -2.34E-05 | 0.000155 | 0.88 |
| **rs798489** | T | C | 7 | -0.035 | 0.0039 | 5.40E-19 | -0.000157 | 0.000166 | 0.34 |
| **rs806794** | G | A | 6 | -0.045 | 0.0038 | 2.30E-31 | -0.000345 | 0.000162 | 0.034 |
| **rs849141** | G | A | 7 | -0.032 | 0.0038 | 1.70E-16 | 0.0001945 | 0.000162 | 0.23 |
| **rs912057** | G | A | 6 | -0.028 | 0.0036 | 3.70E-15 | 9.93E-05 | 0.00015 | 0.51 |
| **rs9388766** | C | T | 6 | -0.026 | 0.0037 | 1.60E-12 | -7.63E-05 | 0.000158 | 0.630001 |
| **rs9890032** | G | C | 17 | -0.025 | 0.0036 | 1.60E-12 | -0.000134 | 0.00015 | 0.37 |
| **rs9993613** | G | T | 4 | -0.027 | 0.0045 | 6.80E-10 | 8.74E-05 | 0.000146 | 0.55 |

Chr: Chromosome.
