# Supplementary material for: Obesity-related indicators and tuberculosis: A Mendelian randomization study
Source: PLoS One. 2024 Apr 1;19(4):e0297905. doi: 10.1371/journal.pone.0297905 (PMC10984409; doi:10.1371/journal.pone.0297905)
Supplement: S4 Table — (DOCX) [file pone.0297905.s005.docx]

**S4 Table: Harmonized dataset of Mendelian randomization for the effect of waist circumference on respiratory tuberculosis.**

| **SNP** | **Effect allele** | **Other allele** | **Chr** | **Exposure** | | | **Outcome** | | |
| --- | --- | --- | --- | --- | --- | --- | --- | --- | --- |
|  |  |  |  | ***β*** | **SE** | ***p*** | ***β*** | **SE** | ***p*** |
| **rs10041657** | A | G | 5 | 0.025 | 0.0038 | 9.10E-11 | -6.36E-05 | 0.000173 | 0.709999 |
| **rs10923712** | A | G | 1 | 0.036 | 0.0033 | 2.60E-27 | 1.10E-05 | 0.000149 | 0.94 |
| **rs1108472** | T | C | 10 | 0.019 | 0.0033 | 1.20E-08 | 0.0002401 | 0.000147 | 0.1 |
| **rs11144688** | A | G | 9 | -0.034 | 0.0059 | 4.50E-09 | 0.0001819 | 0.000226 | 0.42 |
| **rs11205277** | G | A | 1 | 0.026 | 0.0036 | 2.60E-13 | 0.0001601 | 0.000147 | 0.28 |
| **rs1144** | C | T | 7 | 0.019 | 0.0035 | 3.10E-08 | 8.88E-05 | 0.000152 | 0.56 |
| **rs12317176** | C | T | 12 | -0.021 | 0.0034 | 3.90E-10 | 7.97E-05 | 0.000154 | 0.6 |
| **rs12330322** | T | C | 3 | -0.022 | 0.0039 | 1.00E-08 | -4.86E-05 | 0.000175 | 0.780001 |
| **rs12493901** | A | G | 3 | -0.02 | 0.0033 | 9.20E-10 | 0.0001051 | 0.000148 | 0.48 |
| **rs12608504** | G | A | 19 | -0.02 | 0.0034 | 2.80E-09 | -5.79E-05 | 0.000151 | 0.7 |
| **rs12679556** | G | T | 8 | 0.025 | 0.0037 | 9.70E-12 | -5.31E-06 | 0.000168 | 0.97 |
| **rs12700664** | T | C | 7 | -0.018 | 0.0034 | 3.50E-08 | 4.14E-06 | 0.000146 | 0.98 |
| **rs12991495** | C | T | 2 | -0.026 | 0.0037 | 5.80E-13 | 6.12E-05 | 0.000158 | 0.7 |
| **rs1344674** | G | A | 3 | 0.024 | 0.0032 | 7.50E-14 | 0.0002191 | 0.000147 | 0.14 |
| **rs1482852** | G | A | 3 | -0.026 | 0.0034 | 3.70E-14 | -4.14E-06 | 0.000149 | 0.98 |
| **rs16957304** | G | A | 16 | -0.059 | 0.011 | 2.50E-08 | -0.000114 | 0.000344 | 0.74 |
| **rs1776897** | T | G | 6 | -0.058 | 0.0061 | 1.30E-21 | -0.000438 | 0.00025 | 0.079001 |
| **rs1884897** | G | A | 20 | -0.031 | 0.0034 | 9.50E-20 | -0.000122 | 0.000151 | 0.42 |
| **rs2052670** | G | A | 2 | 0.02 | 0.0034 | 8.20E-09 | 0.0002464 | 0.000155 | 0.11 |
| **rs2062708** | C | T | 5 | 0.026 | 0.0045 | 1.00E-08 | -0.000115 | 0.000198 | 0.56 |
| **rs2124969** | C | T | 2 | 0.02 | 0.0034 | 1.20E-09 | -0.000131 | 0.000149 | 0.38 |
| **rs2197271** | G | C | 4 | 0.027 | 0.0038 | 3.40E-13 | 0.0001604 | 0.000167 | 0.34 |
| **rs2214442** | G | A | 7 | 0.026 | 0.0045 | 3.90E-09 | 2.92E-06 | 0.000146 | 0.98 |
| **rs2274432** | A | G | 1 | 0.024 | 0.0035 | 2.40E-12 | -5.94E-05 | 0.000153 | 0.7 |
| **rs2294239** | G | A | 22 | -0.019 | 0.0034 | 2.00E-08 | 4.06E-05 | 0.000148 | 0.780001 |
| **rs2638953** | C | G | 12 | 0.023 | 0.0035 | 2.80E-11 | -9.25E-05 | 0.000156 | 0.55 |
| **rs2745353** | T | C | 6 | 0.029 | 0.0032 | 5.10E-20 | 3.27E-05 | 0.000145 | 0.82 |
| **rs3764419** | A | C | 17 | -0.02 | 0.0034 | 5.50E-09 | -0.000139 | 0.00015 | 0.35 |
| **rs3791679** | G | A | 2 | -0.036 | 0.0038 | 3.90E-21 | -0.000359 | 0.000174 | 0.039 |
| **rs4246302** | G | A | 15 | 0.021 | 0.0036 | 3.90E-09 | -5.84E-05 | 0.000159 | 0.709999 |
| **rs4369779** | C | T | 18 | 0.041 | 0.004 | 1.40E-24 | 0.0002094 | 0.000178 | 0.24 |
| **rs4542783** | C | T | 19 | -0.022 | 0.0039 | 2.20E-08 | -0.000432 | 0.000147 | 0.0032 |
| **rs4567683** | G | A | 15 | -0.023 | 0.0037 | 1.30E-09 | -0.000116 | 0.000165 | 0.48 |
| **rs473902** | G | T | 9 | -0.049 | 0.007 | 1.30E-12 | 3.84E-05 | 0.000261 | 0.88 |
| **rs4886782** | A | G | 15 | -0.024 | 0.0035 | 4.20E-12 | 0.0001091 | 0.000154 | 0.48 |
| **rs606452** | C | A | 11 | -0.026 | 0.0046 | 8.80E-09 | 0.0001942 | 0.000206 | 0.35 |
| **rs6556301** | T | G | 5 | 0.026 | 0.0039 | 1.40E-11 | 4.96E-05 | 0.000152 | 0.74 |
| **rs6751657** | C | T | 2 | 0.019 | 0.0032 | 4.50E-09 | 4.38E-05 | 0.000146 | 0.760001 |
| **rs6900530** | T | C | 6 | -0.055 | 0.0091 | 1.60E-09 | 0.000261 | 0.000419 | 0.53 |
| **rs7162542** | G | C | 15 | 0.037 | 0.0033 | 1.20E-28 | -0.000223 | 0.000148 | 0.13 |
| **rs7430034** | T | C | 3 | 0.02 | 0.0034 | 4.80E-09 | 6.28E-05 | 0.000151 | 0.68 |
| **rs754133** | A | G | 12 | 0.029 | 0.0035 | 4.00E-17 | -0.000194 | 0.000153 | 0.2 |
| **rs7684221** | A | G | 4 | -0.025 | 0.0045 | 2.10E-08 | 1.11E-05 | 0.000196 | 0.95 |
| **rs7689420** | C | T | 4 | 0.029 | 0.0044 | 1.40E-11 | 0.0002689 | 0.000193 | 0.16 |
| **rs7727544** | T | C | 5 | 0.02 | 0.0033 | 2.30E-09 | -3.86E-06 | 0.000147 | 0.98 |
| **rs7801581** | T | C | 7 | 0.026 | 0.004 | 1.40E-10 | -0.00019 | 0.00017 | 0.26 |
| **rs780159** | G | A | 10 | 0.019 | 0.0034 | 1.60E-08 | 0.0003534 | 0.00015 | 0.019 |
| **rs798502** | C | A | 7 | -0.024 | 0.0036 | 2.90E-11 | -0.000146 | 0.00016 | 0.36 |
| **rs806794** | G | A | 6 | -0.028 | 0.0036 | 5.70E-15 | -0.000345 | 0.000162 | 0.034 |
| **rs822531** | T | C | 7 | 0.024 | 0.0044 | 2.60E-08 | 0.0001718 | 0.000183 | 0.35 |
| **rs849140** | C | T | 7 | -0.028 | 0.0033 | 5.70E-17 | 7.38E-05 | 0.000147 | 0.62 |
| **rs882367** | T | C | 17 | -0.025 | 0.0035 | 1.50E-13 | -9.76E-05 | 0.000154 | 0.53 |
| **rs9389986** | A | T | 6 | -0.024 | 0.0037 | 3.30E-11 | 3.50E-05 | 0.000161 | 0.83 |
| **rs9435732** | T | C | 1 | -0.031 | 0.0037 | 7.60E-17 | -0.000134 | 0.000165 | 0.42 |
| **rs991967** | C | A | 1 | 0.026 | 0.0035 | 2.90E-13 | 0.000157 | 0.00016 | 0.33 |

Chr: Chromosome.
