# Supplementary material for: Obesity-related indicators and tuberculosis: A Mendelian randomization study
Source: PLoS One. 2024 Apr 1;19(4):e0297905. doi: 10.1371/journal.pone.0297905 (PMC10984409; doi:10.1371/journal.pone.0297905)
Supplement: S5 Table — (DOCX) [file pone.0297905.s006.docx]

**S5 Table: Multivariate MR analysis of BMI and smoking, type 2 diabetes and educational attainment.**

| **Exposure** | **Outcome** | **OR** | **95%CI** | ***p*-value** |
| --- | --- | --- | --- | --- |
| Type 2 diabetes | Respiratory tuberculosis | 0.984 | 0.830-1.166 | 0.850 |
| Educational attainment | Respiratory tuberculosis | 0.408 | 0.044-3.791 | 0.431 |
| Smoking | Respiratory tuberculosis | 2.180 | 0.328-14.445 | 0.420 |
| Waist-to-hip ratio | Respiratory tuberculosis | 0.590 | 0.327-1.065 | 0.080 |
