# Supplementary material for: Obesity-related indicators and tuberculosis: A Mendelian randomization study
Source: PLoS One. 2024 Apr 1;19(4):e0297905. doi: 10.1371/journal.pone.0297905 (PMC10984409; doi:10.1371/journal.pone.0297905)
Supplement: S6 Table — (DOCX) [file pone.0297905.s007.docx]

**S6 Table: Multivariate MR analysis of waist circumference and smoking, type 2 diabetes and educational attainment.**

| **Exposure** | **Outcome** | **OR** | **95%CI** | ***p*-value** |
| --- | --- | --- | --- | --- |
| Type 2 diabetes | Respiratory tuberculosis | 1.023 | 0.865-1.210 | 0.792 |
| Educational attainment | Respiratory tuberculosis | 0.305 | 0.053-1.760 | 0.184 |
| Smoking | Respiratory tuberculosis | 0.941 | 0.192-4.602 | 0.940 |
| Waist circumference | Respiratory tuberculosis | 2.301 | 1.211-4.372 | **0.011** |
