# Supplementary material for: Obesity-related indicators and tuberculosis: A Mendelian randomization study
Source: PLoS One. 2024 Apr 1;19(4):e0297905. doi: 10.1371/journal.pone.0297905 (PMC10984409; doi:10.1371/journal.pone.0297905)
Supplement: S7 Table — (DOCX) [file pone.0297905.s008.docx]

**S7 Table: Multivariate MR analysis of hip circumference and smoking, type 2 diabetes and educational attainment.**

| **Exposure** | **Outcome** | **OR** | **95%CI** | ***p*-value** |
| --- | --- | --- | --- | --- |
| Type 2 diabetes | Respiratory tuberculosis | 1.050 | 0.886-1.245 | 0.571 |
| Educational attainment | Respiratory tuberculosis | 0.413 | 0.073-2.333 | 0.317 |
| Smoking | Respiratory tuberculosis | 1.380 | 0.288-6.608 | 0.687 |
| Hip circumference | Respiratory tuberculosis | 1.438 | 0.849-2.433 | 0.176 |
