# Supplementary material for: Obesity-related indicators and tuberculosis: A Mendelian randomization study
Source: PLoS One. 2024 Apr 1;19(4):e0297905. doi: 10.1371/journal.pone.0297905 (PMC10984409; doi:10.1371/journal.pone.0297905)
Supplement: S8 Table — (DOCX) [file pone.0297905.s009.docx]

**S8 Table: Multivariate MR analysis of waist-to-hip ratio and smoking, type 2 diabetes and educational attainment.**

| **Exposure** | **Outcome** | **OR** | **95%CI** | ***p*-value** |
| --- | --- | --- | --- | --- |
| Type 2 diabetes | Respiratory tuberculosis | 1.044 | 0.880-1.238 | 0.621 |
| Educational attainment | Respiratory tuberculosis | 0.337 | 0.058-1.948 | 0.224 |
| Smoking | Respiratory tuberculosis | 2.086 | 0.351-8.053 | 0.516 |
| Waist-to-hip ratio | Respiratory tuberculosis | 0.014 | 0.170-1.014 | 0.054 |
