# Supplementary material for: Obesity-related indicators and tuberculosis: A Mendelian randomization study
Source: PLoS One. 2024 Apr 1;19(4):e0297905. doi: 10.1371/journal.pone.0297905 (PMC10984409; doi:10.1371/journal.pone.0297905)
Supplement: Attachment — Submitted filename: Review.docx [file pone.0297905.s010.docx]

**Summary:**

This is a two-sample Mendelian randomization study making use of publicly available genetic association summary statistics to investigate causal relationships between anthropometric traits and tuberculosis risk. The authors identify consistent evidence for a causal relationship between waist-circumference and TB, and possible evidence for a causal relationship between hip-circumference and TB.

The statistical design and reporting of the study is overall done well. While I am not aware of any similar studies, I am not thoroughly acquainted with the applied Mendelian randomization literature in the tuberculosis space.

**Major Remarks:**

1. The reporting of causal effect estimates from Mendelian randomization should be chosen from a method identified as most appropriate for the data. In this case, I would recommend reporting the IVW estimate and P-value as the primary finding unless other methods suggest a presence of horizontal pleiotropy or sensitivity analyses show evidence of heterogeneity. This is consistent with the author’s method section suggesting that the IVW method is preferred and will be the primary focus. (Lines 117-118 & Line 133-134) Therefore, the results section should be framed as reporting the IVW estimate foremost unless Table 3 shows substantial evidence of heterogeneity for the outcome or the pleiotropy robust methods (median, PRESSO) produce substantially different effect sizes.

Based on this, for the waist circumference outcome I would recommend highlighting the effect size and P-Value from the IVW method primarily, and commenting that no heterogeneity was observed and that pleiotropy robust methods like weighted median and PRESSO provided similar effect sizes.

2. The hip-circumference results differ between those reported in text on lines 166-170 versus Table 2. Please clarify which set of results are the actual results for the hip-circumference outcome. If the table results are accurate, please explain why the MR-PRESSO method alone should categorize hip-circumference as a significant finding while no evidence of heterogeneity or pleiotropy is observed in Table 3 along with no difference in effect sizes for the IVW (1.49) and robust methods (Median=1.50, PRESSO = 1.50). Additionally, the substantial increase in MR-Egger effect size should be explained.
3. For those outcomes being reported as significantly causally associated with TB, it should be explained why the Mode & Weighted Mode methods produce substantially larger effect sizes and P-Values than the other methods.

**Minor Remarks:**

1. It should be made clear that the IVW method relies on the assumptions of MR for unbiased estimation, while the other five methods (medians, modes, & PRESSO) all attempt to correct for violations of the exclusion assumption.
2. More detail should be provided in the main text about the resources used for instrument selection and the corresponding association studies for the exposures and outcome.
3. The leave-one-out analysis is not sufficiently explained in the methods and it is hard for me to interpret the results without the supplementary figures.

Minor line-by-line suggestions:

Line 60: The last sentence of the second paragraph in the introduction seems contradictory. The authors state that low BMI is a known risk factor for TB, the subsequently state that BMI TB associations are controversial. Some clarification or expanded explanation here may be useful.

Line 67: MR is not necessarily a whole-genome sequence (WGS) technique.

Line 70: High correlation with the exposure does not ensure that no other confounding factors are present. MR relies on the randomization of alleles during meiosis to remove instrumental variables from confounding effects.

Line 77: Sequencing data… Are these studies WGS?

Line 85: More details provided on the exposure GWAS should be provided.

Line 90: More details on the LD exclusion should be provided. Was pairwise LD computed for each instrument, and if so, what resource was used.

Line 95: Provide details on F-Statistic computation. Additionally, given the large sample sizes, F-statistics may not be very informative.

Line 105: Sequenced… Was the data obtained from WGS study? More details on the outcome GWAS would be helpful.

Line 106: What is meant by the point at which IVs intersected with the exposure data? And how was it incorporated into the MR methods?

Line 118: Citation for IVW validation efficacy?

Line 119: A justification for 0.05 as a significance threshold may be helpful given the 6 tests for each outcome.

Line 145-147: With such large sample sizes, large F-statistics are not surprising and may not give much insight into instrument strength.

Line 173: positive *causal* relationships

Line 176: Non-significant findings do not necessarily suggest a lack of a causal relationship but may be due to lack of statistical power or sufficient instruments to identify a true causal relationship.

Line 202: indicators and TB using *two-sample mendelian randomization.*

Line 202-205: These MR methods provide linear estimates of effect, not correlations.
