# Supplementary material for: Obesity-related indicators and tuberculosis: A Mendelian randomization study
Source: PLoS One. 2024 Apr 1;19(4):e0297905. doi: 10.1371/journal.pone.0297905 (PMC10984409; doi:10.1371/journal.pone.0297905)
Supplement: Attachment — Submitted filename: Response to Reviewers.docx [file pone.0297905.s011.docx]

Dear PhD. Chunyu Liu,
Thank you for your letter and for the reviewers’ comments concerning our manuscript entitled “Obesity-related indicators and tuberculosis a Mendelian randomization study” (ID: PONE-D-23-09863). Those comments are all valuable and very helpful for revising and improving our paper, as well as the important guiding significance to our researches. We have studied comments carefully and have made correction which we hope meet with approval. In tracking the revised manuscript, we yellow the revised and supplemented parts.

We have fully addressed each concern and hope that this revised manuscript is now acceptable. The main corrections in the paper and the responds to the reviewer’s comments are as following:

**Responds to the Journal requirements:**

1. **Response to comment:** Please ensure that your manuscript meets PLOS ONE's style requirements, including those for file naming.

**Response:** Thank you for the reminder. We have named the submitted files as requested and the format of the manuscript has been revised as requested.

2. **Response to comment:** Thank you for stating the following financial disclosure:
"This study was supported by the impact of 4C continuity of care model on non-invasive ventilator use in patients with chronic obstructive pulmonary disease (Project number: 2101320712A2012). "

Please state what role the funders took in the study.  If the funders had no role, please state: "The funders had no role in study design, data collection and analysis, decision to publish, or preparation of the manuscript."
If this statement is not correct you must amend it as needed.
Please include this amended Role of Funder statement in your cover letter; we will change the online submission form on your behalf.

**Response:** The funders had no role in study design, data collection and analysis, decision to publish, or preparation of the manuscript.

**Responds to the Reviewer’s comments:**

**Reviewer #1:**

1. **Response to comment:** Please all the obesity indicators in the abstract to facilitate readers.

**Response:** Thank you for your suggestions for this article. We have added obesity-related indicators in the second paragraph of the introduction section. The additions are as follows: In clinical practice, obesity-related indicators include body mass index (BMI), waist circumference, BMI-adjusted waist-to-hip ratio, hip circumference, arm circumference, blood lipid level, fat mass and low lean body mass. **(Page. 3, Line. 56-59)**

2. **Response to comment:** Line 43, is M. tuberculosis the abbreviation of Mycobacterium tuberculosis? Please specify.

**Response:** We apologize for the inconvenience of reading this article due to an oversight on our part. We have made a correction to add the full name of Mycobacterium tuberculosis. **(Page. 3, Line. 42)**

3. **Response to comment:** Please polish the language and transition between paragraphs. For example, Line 52, it seems too sudden jumping from the first paragraph to the second paragraph; Line 59, two “however”s in two sentences; etc.

**Response:** Thanks to your valuable comments, we have made changes to the overall language of the article. As you pointed out in the paragraph-to-paragraph connection and sentence-to-sentence conjunction, we made changes.

4. **Response to comment:** Line 72, the second assumption of MR seems not correct. There is no assumption that IVs should be uncorrelated with each other. And the authors mentioned these three assumptions again in the IV selection part, and the second assumption is a different one there.

**Response:** Your suggestions are very valuable. We apologize for the lack of clarity due to an oversight on our part. The second assumption of Mendelian randomization should be that IVs are independent of confounding factors and exhibit independence of instrumental variables. Therefore, we have made the following changes in the text: Secondly, these IVs should not act on the outcome through confounding factors to ensure that they are independent. **(Page. 4, Line. 75-76)**

5. **Response to comment:** Line 85, the citation of GIANT BMI GWAS is from 2013 paper, but as far as I know, the Locke et al. 2015 is the GIANT BMI GWAS paper that corresponds to the GWAS summary stats on GIANT website (https://portals.broadinstitute.org/collaboration/giant/index.php/GIANT_consortium_data_files#GWAS_Anthropometric_2015_BMI_Summary_Statistics). Can you check which GWAS summary stats was actually used in the paper?

**Response:** Thank you very much for the tip, and we are sorry that we have not been very strict in correcting the references. As you said, we made the change to cite the correct reference, and we cited Genetic studies of body mass index yield new insights for obesity biology (PMID: 25673413). **(Page. 5, Line. 88)**

6. **Response to comment:** Line 86, is waist-hip-ratio the actual exposure used? Or waist-hip-ratio adjusted for BMI?

**Response:** Thank you for your comments. The waist-to-hip ratio selected for this study is from the Dataset: ieu-a-72, and the data provided are for the waist-to-hip ratio, not for the BMI adjusted.

7. **Response to comment:** Line 108, what is genetic data point?

**Response:** We are very sorry that we did not express ourselves clearly. We have changed genetic data point to SNPs. **(Page. 6, Line. 124)**

8. **Response to comment:** The authors first present the main analyses using six methods, then go to Heterogeneity and horizontal multiplicity tests and leave-one-out analyses as sensitivity analyses. But from my understanding, the logic should be first check heterogeneity and pleiotropy, if there is any IVs with pleiotropy (can be identified by MR-PRESSO individual test), then remove those problematic IVs and perform the primary MR analyses. So I would suggest restructure the results section to check heterogeneity and pleiotropy -> primary MR analyses -> leave one out as sensitivity analysis.

**Response:** Thank you for your suggestion, and we think it is very valuable. Based on your valuable suggestions, we have adjusted the order of the results section, as well as the order of Table 2 and Table 3. The result section is modified as follows: the second part of the result is modified to **Heterogeneity and horizontal multiplicity tests**, and the fourth part is added to **Sensitivity analysis**. **(Page. 9, Line. 182-188; Page. 12-13, Line. 214-222)**

9. **Response to comment:** In Table 2, sometimes results are different between different methods, for example, for waist circumference, it is significant in IVW but not significant in MR egger. May consider discuss those discrepancies in the discussion section.

**Response:** Your advice is very valuable. We have added in the discussion section to explain this situation. The additions are as follows: On the other hand, in the leave-one-out test, we found that there are some IVs that affect the overall effect, which may be due to the more complex risk factors of TB and the presence of some potential influences. This leads to the results of IVW suggesting a significant causal effect, but the results of other complementary tests such as MR egger, Simple and Weighted mode have larger p-values. **(Page. 13-14, Line. 236-240)**

10. **Response to comment:** We need higher resolution for all the figures.

**Response:** We are very sorry that the clarity of our previous pictures was not adjusted properly. We have adjusted all the images according to your valuable suggestions and modified the clarity of all the figures. **(Figure 1, 2, 3 and Supplementary Figure 1)**

**Reviewer #2:**

This is a two-sample Mendelian randomization study making use of publicly available genetic association summary statistics to investigate causal relationships between anthropometric traits and tuberculosis risk. The authors identify consistent evidence for a causal relationship between waist-circumference and TB, and possible evidence for a causal relationship between hip-circumference and TB.

The statistical design and reporting of the study is overall done well. While I am not aware of any similar studies, I am not thoroughly acquainted with the applied Mendelian randomization literature in the tuberculosis space.

**Response to comment:** Thank you very much for reviewing our manuscript, your suggestions are all very valuable. In particular, thank you for your careful review of our manuscript and for guiding us to a deeper understanding of Mendelian randomization studies. Thank you again for your hard work.

Major Remarks:

1. **Response to comment:** The reporting of causal effect estimates from Mendelian randomization should be chosen from a method identified as most appropriate for the data. In this case, I would recommend reporting the IVW estimate and P-value as the primary finding unless other methods suggest a presence of horizontal pleiotropy or sensitivity analyses show evidence of heterogeneity. This is consistent with the author’s method section suggesting that the IVW method is preferred and will be the primary focus. (Lines 117-118 & Line 133-134) Therefore, the results section should be framed as reporting the IVW estimate foremost unless Table 3 shows substantial evidence of heterogeneity for the outcome or the pleiotropy robust methods (median, PRESSO) produce substantially different effect sizes.

Based on this, for the waist circumference outcome I would recommend highlighting the effect size and P-Value from the IVW method primarily, and commenting that no heterogeneity was observed and that pleiotropy robust methods like weighted median and PRESSO provided similar effect sizes.

**Response:** Thank you very much for your advice, which is very professional. Based on your valuable suggestions we have redescribed in the results section, mainly for IVW, the rest of the test is supplementary to the description of the role. The modifications we have made are as follows: MR-PRESSO suggests no heterogeneity of results **(****Page. 11, Line. 198)**; the results of weighted median, MR Egger and MR-PRESSO suggest no heterogeneity and validate a similar trend effect to the IVW test (**Page. 11, Line. 201-202**)

2. **Response to comment:** The hip-circumference results differ between those reported in text on lines 166-170 versus Table 2. Please clarify which set of results are the actual results for the hip-circumference outcome. If the table results are accurate, please explain why the MR-PRESSO method alone should categorize hip-circumference as a significant finding while no evidence of heterogeneity or pleiotropy is observed in Table 3 along with no difference in effect sizes for the IVW (1.49) and robust methods (Median=1.50, PRESSO = 1.50). Additionally, the substantial increase in MR-Egger effect size should be explained.

**Response:** Thank you for your suggestions. We are very, very sorry for the problem in presenting the results for hip circumference in Table 3(we modify the original Table 2 to Table 3). The results presented in Table 3 are the results of our preliminary MR analysis, based on which we removed some of the SNPs related to confounding factors as described in the text, but due to an oversight there was an editing error in Table 3. We re-edited the data in Table 3, and the data depicted in the second part of the results in the text are correct. This problem is very serious and we apologize again.

3. **Response to comment:** For those outcomes being reported as significantly causally associated with TB, it should be explained why the Mode & Weighted Mode methods produce substantially larger effect sizes and P-Values than the other methods.

**Response:** Your advice is very valuable. Based on the results we got, we have analyzed them and explained them in the discussion section. The additions are as follows: On the other hand, in the leave-one-out test, we found that there are some IVs that affect the overall effect, which may be due to the more complex risk factors of TB and the presence of some potential influences. This leads to the results of IVW suggesting a significant causal effect, but the results of other complementary tests such as MR egger, Simple and Weighted mode have larger p-values. **(Page. 13-14, Line. 236-240)**

Minor Remarks:

1. **Response to comment:** It should be made clear that the IVW method relies on the assumptions of MR for unbiased estimation, while the other five methods (medians, modes, & PRESSO) all attempt to correct for violations of the exclusion assumption.

**Response:** Thank you very much for your guidance. Based on your valuable suggestions we have added a description of the six methods of MR analysis examination in the manuscript in the discussion section. The additions are as follows: We used the results of IVW as a criterion for judging the causal relationship between exposure and outcome, which relied on unbiased estimates of the three assumptions of the MR analysis, distinguishing it from the other five tests, which mainly assessed the correction of the exclusion assumption. **(Page. 13, Line. 227-230)**

2. **Response to comment:** More detail should be provided in the main text about the resources used for instrument selection and the corresponding association studies for the exposures and outcome.

**Response:** Thank you for your valuable advice. We have added a description of exposure and outcomes and details of screening for IVs in the Materials and Methods section. The additions are as follows:

(1) BMI (unit: kg/m^2) was selected from the ieu-a-785 dataset, containing 152893 samples, and the sequencing results of 2477659 SNPs. Waist circumference (unit: cm) was selected from the ieu-a-66 dataset, containing 245,756 samples, and the sequencing results of 254,7573 SNPs. Hip circumference (unit: cm) was selected from the ieu-a-54 dataset, containing 225487 samples, and the sequencing results of 2542663 SNPs. Waist-hip ratio was selected from the ieu-a-72 dataset, containing 224459 samples, and sequencing results of 2562516 SNPs. **(Page. 5, Line. 90-97)**
(2) The screening criteria were p < 5×10^-8^, distance of linkage disequilibrium (LD) > 10000 kb and r^2^ < 0.001. In our screening criteria, kb represents the length of the region between the LDs considered, while r^2^ = 1 represents a complete LD relationship between two SNPs, and r^2^ = 0 represents a complete linkage equilibrium. **(Page. 5, Line. 98-102)**
(3) In this study, the finn-b-TBC_RESP dataset was selected from the GWAS database as the outcome data. This dataset contained 849 TB patients as well as 217632 control samples, with a total of 16380466 SNPs detected. **(Page. 6, Line. 119-121)**

3. **Response to comment:** The leave-one-out analysis is not sufficiently explained in the methods and it is hard for me to interpret the results without the supplementary figures.

**Response:** Thank you for your valuable advice. We added an explanation of the leave-one-out method in paragraph Sensitivity test of two-sample MR of the Material and methods section and added a figure of the results of the leave-one-out method in Figure 3. The additions are as follows: The leave-one-out method involves gradually eliminating each SNP, calculating the meta-effects of the remaining SNPs, and observing whether changes occur after eliminating each SNP. **(Page. 7, Line. 146-148; Figure 3)**

Minor line-by-line suggestions:

1. **Response to comment:** Line 60: The last sentence of the second paragraph in the introduction seems contradictory. The authors state that low BMI is a known risk factor for TB, the subsequently state that BMI TB associations are controversial. Some clarification or expanded explanation here may be useful.

**Response:** Your advice is very helpful. Based on your valuable suggestions, we have revised the description in the second paragraph of the introduction. Our intention was to express that previous studies have shown that low body mass index is a high risk factor for tuberculosis, but the risk of high body mass index for tuberculosis is controversial; therefore, we wanted to further investigate the effect of obesity on the risk of developing tuberculosis.

We have made the following changes in the manuscript: This is quite different from p0revious studies, which have shown that being underweight or having a low body mass index (BMI) is a risk factor for active TB. However, the evidence on the effect of obesity on tuberculosis is not well documented, and the association between low BMI, which is a known risk factor for TB, and high BMI or other obesity-related indicators, including waist circumference and hip circumference, and the risk of TB is controversial. **(Page. 3-4, Line. 61-66)**

2. **Response to comments:** Line 67: MR is not necessarily a whole-genome sequence (WGS) technique.

**Response:** Thank you very much for your valuable advice and your advice is very helpful. Our description's inaccurate. In the manuscript we revised the following: Mendelian randomization (MR) analysis utilizes single nucleotide polymorphisms (SNPs) in whole genome sequencing data as instrumental variables (IVs). **(Page. 4, Line. 68-70)**

3. **Response to comments:** Line 70: High correlation with the exposure does not ensure that no other confounding factors are present. MR relies on the randomization of alleles during meiosis to remove instrumental variables from confounding effects.

**Response:** Thank you very much for pointing out our flaws. Based on your valuable comments, we have redescribed the hypothetical principle of Mendelian randomization. The modifications are as follows: Moreover, MR analysis has the advantage of relying on the randomization of alleles during meiosis to eliminate the confounding effect of instrumental variables. MR analysis relies on requisite basic assumptions, the first of which is that IVs should be highly correlated with exposure factors. Secondly, these IVs should not act on the outcome through confounding factors to ensure that they are independent. **(Page. 4, Line. 71-76)**

4. **Response to comments:** Line 77: Sequencing data… Are these studies WGS?

**Response:** Thanks for your comment. We would like to express that the GWAS database contains a wide range of populations with different characteristics and the corresponding whole genome sequencing data and that the database is useful for large scale population studies. We have modified the text as follows: In addition, the Genome-Wide Association Study (GWAS) database provides a large amount of whole-genome sequencing data for different characteristics of the population, which is more conducive to large-scale experimental studies. **(Page. 4, Line. 89-92)**

5. **Response to comments:** Line 85: More details provided on the exposure GWAS should be provided.

**Response:** Your advice is very important. Based on your suggestions, we have supplemented the description of the exposure data with the following additions: BMI (unit: kg/m^2) was selected from the ieu-a-785 dataset, containing 152893 samples, and the sequencing results of 2477659 SNPs. Waist circumference (unit: cm) was selected from the ieu-a-66 dataset, containing 245,756 samples, and the sequencing results of 254,7573 SNPs. Hip circumference (unit: cm) was selected from the ieu-a-54 dataset, containing 225487 samples, and the sequencing results of 2542663 SNPs. Waist-hip ratio was selected from the ieu-a-72 dataset, containing 224459 samples, and sequencing results of 2562516 SNPs. **(Page. 5, Line. 90-97)**

6. **Response to comment:** Line 90: More details on the LD exclusion should be provided. Was pairwise LD computed for each instrument, and if so, what resource was used.

**Response:** Your suggestion is very helpful. The MR method to calculate LD is achieved by the adjustment of two parameters kd and r^2^ in the TwoSampleMR package. Therefore, we have described them in detail in the manuscript, with the following additions: The screening criteria were p < 5×10^-8^, distance of linkage disequilibrium (LD) > 10000 kb and r^2^ < 0.001. In our screening criteria, kb represents the length of the region between the LDs considered, while r^2^ = 1 represents a complete LD relationship between two SNPs, and r^2^ = 0 represents a complete linkage equilibrium. **(Page. 5, Line. 98-102)**

7. **Response to comment:** Line 95: Provide details on F-Statistic computation. Additionally, given the large sample sizes, F-statistics may not be very informative.

**Response:** Your advice is very helpful. We supplemented the manuscript with the formula for the F-statistic with the following additions: F = (N-k-1)/k × R^2^/(1-R^2^), where N represents the sample size in the exposed data, k is the number of IVs, and R^2^ is the coefficient of determination. **(Page. 5-6, Line. 107-109)**

8. **Response to comment:** Line 105: Sequenced… Was the data obtained from WGS study? More details on the outcome GWAS would be helpful.

**Response:** Your reminder is very valuable and helpful to us. The SNPs for the endings we used were all from the GWAS database. Based on your suggestion, we have added to the description of the outcome data with the following modifications: In this study, the finn-b-TBC_RESP dataset was selected from the GWAS database as the outcome data. This dataset contained 849 TB patients as well as 217632 control samples, with a total of 16380466 SNPs detected. **(Page. 6, Line. 119-123)**

9. **Response to comment:** Line 106: What is meant by the point at which IVs intersected with the exposure data? And how was it incorporated into the MR methods?

**Response:** Your correction is very meaningful. What we describe is to use the IVs of the exposed data to take the intersection in the outcomes data, and the SNPs after taking the intersection as the final IVs for MR analysis. We are sorry that our previous description was inaccurate. The modifications we made are as follows: In addition, the SNPs of the IVs as exposure factors were intersected with the outcome data and used as the final SNPs for performing MR analysis. **(Page. 6, Line. 121-123)**

10. **Response to comment:** Line 118: Citation for IVW validation efficacy?

**Response:** Thanks for your professional reminder. Based on your valuable suggestions we have added the corresponding references PMID: 23863760 and PMID: 26661904. **(Page. 7, Line. 133)**

11. **Response to comment:** Line 119: A justification for 0.05 as a significance threshold may be helpful given the 6 tests for each outcome.

**Response:** Thank you for reminding me. Based on your valuable suggestions we have added an explanation for the definition of the p-value, which is added as follows: In this study, six tests were calculated for the MR analysis of each exposure factor, and the IVW test results were mainly used as the main result to determine the causal relationship, and the rest of the results could not infer the causal effect for the time being, so this study in the analysis of the results was considered that p<0.05 for IVW was considered to be statistically significant. **(Page. 7, Line. 133-138)**

12. **Response to comment:** Line 145-147: With such large sample sizes, large F-statistics are not surprising and may not give much insight into instrument strength

**Response:** Thanks for your reminding. As you said, the validation efficacy of the F-statistic values may be reduced when the sample is studied. However, we have reviewed a lot of literature and have not been able to find an alternative way to calculate it for the time being. Therefore, at the end of the discussion section, we add the limitations of the validation effectiveness of the F-statistics. We modified the following: At the same time, the data of F-statistic value in the study of large sample data analysis will be very large, but the corresponding validation effectiveness will be reduced. **(Page. 16, Line. 290-292)**

13. **Response to comment:** Line 173: positive causal relationships

**Response:** Your advice is very helpful. Based on your suggestion, we modified it to positive causal relationships. **(Page. 11, Line. 206)**

14. **Response to comment:** Line 176: Non-significant findings do not necessarily suggest a lack of a causal relationship but may be due to lack of statistical power or sufficient instruments to identify a true causal relationship.

**Response:** The explanation you provided is very helpful to us. As you said, we have added an explanation of the negative causality in the text at the end of the first paragraph of the Discussion section. The modification is as follows: It may be due to lack of statistical power or sufficient IVs to identify a true causal relationship. **(Page. 14, Line. 242-243)**

15. **Response to comment:** Line 202: indicators and TB using two-sample mendelian randomization.

**Response:** Your advice is very helpful. Based on your suggestion, we modified it to two-sample MR. **(Page. 13, Line. 227)**

16. **Response to comment:** Line 202-205: These MR methods provide linear estimates of effect, not correlations.

**Response:** Thank you for the reminder. Based on your suggestion, we modified it to linear estimates of effect. **(Page. 13, Line. 232)**

We tried our best to improve the manuscript and made some changes in the manuscript. In addition, We have revised and added to the structure of the results and the discussion section of the manuscript as a whole based on the valuable comments of the reviewers.

We appreciate for Editor and Reviewers’ warm work earnestly, and hope that the correction will meet with approval.

Once again, thank you very much for your comments and suggestions.
