# Supplementary material for: Obesity-related indicators and tuberculosis: A Mendelian randomization study
Source: PLoS One. 2024 Apr 1;19(4):e0297905. doi: 10.1371/journal.pone.0297905 (PMC10984409; doi:10.1371/journal.pone.0297905)
Supplement: Attachment — Submitted filename: Response to Reviewers.docx [file pone.0297905.s012.docx]

Dear PhD. Chunyu Liu,
Thank you for your letter and for the reviewers’ comments concerning our manuscript entitled “Obesity-related indicators and tuberculosis a Mendelian randomization study” (ID: PONE-D-23-09863R1). Those comments are all valuable and very helpful for revising and improving our paper, as well as the important guiding significance to our researches. We have studied comments carefully and have made correction which we hope meet with approval. In tracking the revised manuscript, we yellow the revised and supplemented parts.

We have fully addressed each concern and hope that this revised manuscript is now acceptable. The main corrections in the paper and the responds to the reviewer’s comments are as following:

**Responds to the comments to the Author:**

The above reviewers' comments evaluated the article in terms of content, language, statistical methods, etc. In many aspects, we still have some deficiencies, and we revised the article again, especially thanks to reviewer 3 for his help to us, his suggestions are very valuable and professional, which made us realize the deficiencies of our study.

**Responds to the Reviewer’s comments:**

**Reviewer #2:**

1. **Response to comment:**

Line 52-53: Remove “As we all know”
Lines 133-138: This wording is a bit misleading. remove “The rest of the results could not infer the causal effect for the time being”. Enough to say that IVW was the main method and therefore P<0.05.
Lines 228-230: “unbiased estimates of the three assumptions” does not make sense. The assumptions are not being estimated. How about. “We used the results of IVW as a criterion for judging
228 the causal relationship between exposure and outcome, while the other methods assessed the validity of the exclusion assumption(32).”

**Response:** Thank you very much for your careful comments, based on your valuable suggestions, we have made changes, thank you again for your hard work.

**Reviewer #3:**

The authors used two-sample MR to infer causal association between obesity-related indicators and TB. They observed a causal relationship between waist circumference and TB, and between hip circumference and TB. While these findings are intriguing, further analyses are necessary to validate the robustness and reliability of the results. However, it is essential to point out that the manuscript contains several instances of inappropriate usage of professional terminology and poorly structured sentences, which may impact the clarity and coherence of the content. Therefore, it is advisable to reevaluate the wording and flow of the paper to ensure its accuracy and comprehensibility.

**Response to comment:** Thank you very much for your comments. We think you are very well versed in the study of MR and your suggestions have given us a better understanding of MR. Your reminder makes us realize that removing confounding factors via PhenoScanner is not accurate enough. In order to minimize the pleiotropy from smoking, type 2 diabetes, and educational attainment, we conducted a multivariable MR analysis with adjustments for genetically predicted of these variables. We have reanalyzed the overall data and have come up with some new conclusions that give more insight into our study. Thanks again for your dedication and guidance.

1. **Response to comment:** The authors tested BMI, waist circumference, hip circumference and waist to hip ratio, it is better to specify a threshold for multiple testing.

**Response:** Your advice is very important. Based on your valuable suggestions we have increased the threshold for multiple testing. We modified it as follows: “Given that our study evaluates four null hypotheses, we have employed a Bonferroni-corrected Type I error rate of αBonf = 0.0125(0.05/4=0.0125) to address the issue of multiple testing” (**Page. 7, Line. 134-136**)

2. **Response to comment:** The authors employed the "PhenoScanner" database to identify and exclude genetic variants associated with potential confounders in their analyses. However, it is crucial to recognize that this approach might not entirely differentiate between horizontal and vertical pleiotropy, as only the former would introduce bias in Mendelian Randomization (MR) studies. Additionally, for many genetic variants, their precise biological functions remain unknown, adding further complexity to the analysis. To enhance the robustness of instrumental variables and strengthen the genetic underpinnings of the study, the authors should consider employing alternative techniques for identifying horizontal pleiotropic single nucleotide polymorphisms (SNPs).

**Response:** Thank you very much for your professional advice. Your reminder makes us realize that removing confounding factors via PhenoScanner is not accurate enough. To make our study more reliable, we supplemented multivariate MR .We modified it as follows: “In order to minimize the pleiotropy from smoking, type 2 diabetes, and educational attainment, we conducted a multivariable MR analysis with adjustments for genetically predicted of these variables.” **(Page. 7, Line. 144-146)**

3. **Response to comment:** The authors accessed tuberculosis (TB) genetic data from a Genome-Wide Association Study (GWAS) database. Unfortunately, the manuscript lacks specific details about the name or source of this GWAS database. In addition, to claim that their analysis is a "two-sample" Mendelian Randomization (MR) analysis, the authors must address whether there is any sample overlapping between the TB genetic data obtained from the GWAS database and the sample used for the exposure data. Without this crucial information, the validity of the "two-sample" MR analysis might be compromised. Sample overlap between datasets used in MR analysis can introduce bias and invalidate the results. Hence, the authors should thoroughly investigate and disclose any shared samples or related individuals between the TB genetic data and exposure data. If there is no overlap, they can confidently proceed with the "two-sample" MR analysis, using distinct datasets for the genetic instrument and exposure, enhancing the robustness and reliability of their study. Otherwise, state in discussion as a limitation of this study.

**Response:** Your advice is very valuable. We are very sorry that due to our negligence, we did not provide a detailed introduction of exposure and outcome data. We have added a description of the exposure and outcome databases in the Materials and Methods section and applied the corresponding references. It was clarified that there is no sample overlap between exposure and outcome. We modified it as follows: “In this study, genetic variation data for obesity-related indicators, including BMI, waist circumference, hip circumference, waist-to-hip ratio, were obtained from the Genetic Investigation of Anthropometric Traits (GIANT) Consortium. The genetic variation data associated with TB was obtained from the Finngen biobank. All the data were sourced from distinct cohorts of European ancestry, with exposures and outcomes stemming from different study, devoid of overlap.” **(Page. 5, Line. 86-95)**

4. **Response to comment:** If adopting multiple testing threshold, for example 0.05/4=0.0125, then almost all of the results become null results.

**Response:** Thank you very much for your advice, which is very professional. According to your reminder, when we initially screened the instrumental variables, smoking, diabetes and education level were regarded as the main confounding factors affecting tuberculosis. Therefore, we re-conducted the two-sample MR analysis and finally determined that waist circumference was in line with the p-values corrected by multiple tests.We modified it as follows: “Based on our comprehensive literature search, we have identified three TB-related factors as potential confounding variables that could impact the genuine causal relationship between obesity-related indicators and TB: smoking, type 2 diabetes, and educational attainment.” **(Page. 5, Line. 90-94, Table 3)**

5. **Response to comment:** The number of IVs used for each exposure variable might be relatively small, which could affect the statistical power of the analysis. Power calculation should be included in the analysis.

**Response:** According to your valuable suggestions, we have supplemented the power. We modified it as follows: “The power corresponding to waist circumference in this study was calculated using an online tool (https://shiny.cnsgenomics.com/mRnd/). The relevant parameters are set as follows: type-I error rate is 0.05, R2 is 0.0002, based on the overall sample size, the final power we get is 84%. This result suggest that this study has sufficient power to explore the causal relationship between waist circumference and TB.” (**Page. 13, Line. 226-231**)

6. **Response to comment:** The analysis conducted in the study did not fully address the main hypothesis of the research. There are several strong risk factors associated with both obesity and tuberculosis, which were not adequately accounted for in the analysis. For instance, lower socioeconomic status is known to increase the risk of tuberculosis and may also contribute to higher rates of obesity due to limited access to healthy foods and opportunities for physical activity. Similarly, smoking is a well-established risk factor for tuberculosis and can also impact body weight, potentially leading to obesity or underweight, depending on smoking habits. Furthermore, diabetes is linked to both obesity and an increased risk of tuberculosis due to its impact on the immune response and susceptibility to infections. To comprehensively examine how these factors may affect tuberculosis, the authors need to incorporate more sophisticated methods beyond simply removing the associated single nucleotide polymorphisms (SNPs) from the analysis. Two-step Mendelian Randomization (MR), multivariable MR, or mediation analysis could be valuable approaches to address the influence of these confounding factors. Two-step MR involves first estimating the effects of the risk factors on the exposure variables (e.g., obesity-related indicators) and then using these estimates to assess the effect of the exposure on the outcome (tuberculosis). Multivariable MR allows for simultaneous assessment of multiple exposures while adjusting for potential confounders. Mediation analysis can help explore whether the effects of the exposure variables on tuberculosis are mediated through other risk factors, such as socioeconomic status, smoking, or diabetes.

By incorporating these methods, the authors can gain a more comprehensive understanding of the relationships between obesity-related indicators, potential confounding factors, and tuberculosis. This will enhance the validity and interpretation of the study's findings and provide more insights into the causal pathways between obesity-related indicators and tuberculosis risk.

**Response:** Your suggestion is very valuable to our research. Thank you very much for your guidance. According to your valuable suggestions, we supplemented the multivariate MR Analysis. We were unable to find a suitable dataset for socioeconomic status in the GWAS database, so we substituted educational attainment. We analyzed the effects of confounding factors such as smoking, diabetes, and education level on our findings using multivariate MR. We modified it as follows: “Simultaneously, in the multivariable MR analysis adjusting for genetically predicted potential confounding factors, the results remained steadfast (S5-8 Tables).” (**Page. 9-10, Line. 188-190**)

Minor Remarks:

1. **Response to comment:** Line 114: Sample mode -> simple mode

**Response:** Thank you very much for your reminding, we have corrected it. **(Page. 6, Line. 127)**

2. **Response to comment:** Line 119 is not clear: “In addition, p <0.05 for each test was considered to be statistically different.”

**Response:** Thank you for your valuable advice. We modified it as follows: “Given that our study evaluates four null hypotheses, we have employed a Bonferroni-corrected Type I error rate of αBonf = 0.0125(0.05/4=0.0125) to address the issue of multiple testing. (**Page. 7, Line. 134-136**)

3. **Response to comment:** If the a number is very small, use scientific digits to show the numbers. For example, write 0.00023 as 2.3e-05 rather than 0.000.

**Response:** Thank you very much for reminding us that we have made some adjustments in the writing of numbers in the article.

4. **Response to comment:** The authors should exercise caution in making strong claims about causality derived from observational data throughout the manuscript. While significant genetic associations may indicate a potential for causality, definitive claims of causation should be avoided. Observational studies, including Mendelian Randomization (MR), can provide valuable insights into causal relationships, but they inherently carry limitations that prevent absolute causality conclusions.

**Response:** Thank you for reminding me. Based on your valuable comments, we have revised the article as a whole. According to the analysis results, we found that waist circumference is likely to be potential risk factors for tuberculosis. We modified it as follows: “In summary, we determined a causal relationship between waist circumference and TB using two-sample MR analysis, which suggested that waist circumference is likely to be potential risk factors.” **(Page. 17, Line. 304-306)**

5. **Response to comments:** The term "horizontal multiplicity" is not a standard term in the context of genetics or statistical analyses. I would suggest to change it throughout the manuscript.

**Response:** Thank you very much for pointing out our problem. We changed it throughout the article and replaced it with pleiotropy. **(Page. 9, Line. 182-187)**

6. **Response to comments:** Line 188 is not clear: the author stated there were no abnormal outliers.

**Response:** We are very sorry that we did not express ourselves clearly. We would like to show that there are no extreme outliers for the SNPs selected in this study through the MR-PRESSO results. We'll change this to “Furthermore, the p-values for MR-Egger intercept and MR-PRESSO global test were > 0.05 for the pleiotropy test, indicating that the SNPs included had no pleiotropy and no abnormal outliers were found for the SNPS selected in this study(Table 3).” **(Page. 9, Line. 185-188)**

7. **Response to comments:** A recent MR study reported a causal relationship between waist circumference and type 2 diabetes, which led to an increased risk for development. This sentence appears to be incomplete.

**Response:** We are very sorry that we did not express ourselves clearly due to our negligence. We'll change this to “A recent MR study reported a causal relationship between waist circumference and type 2 diabetes, and type 2 diabetes also leads to an increased risk of TB”. **(Page. 14, Line. 255-257)**

8. **Response to comments:** The MR-egger test for the hip circumference yielded an extremely large OR and a wide confidence interval, how should that be interpreted? Concluding this result had the same trend in effect was not accurate.

**Response:** Your advice is very valuable. As for the problem of too large confidence interval, we included it in the limitations of this study. We believe that it may be related to the selected instrumental variables, and subsequent increases in their number may solve this problem. In addition, following your reminder, we realized that it would be imprecise to conclude that this method has the same trend as other methods, so we removed this conclusion from the manuscript. We'll change this to “In additon, some excessive confidence intervals were obtained in the MR analysis, and the sample size and IVs need to be expanded in the future.”. **(Page. 16, Line. 298-300)**

We tried our best to improve the manuscript and made some changes in the manuscript. In addition, We have revised and added to the structure of the results and the discussion section of the manuscript as a whole based on the valuable comments of the reviewers.

We appreciate for Editor and Reviewers’ warm work earnestly, and hope that the correction will meet with approval.

Once again, thank you very much for your comments and suggestions.
